# The Identification of a Novel Calcium-Dependent Link Between NAD^+^ and Glucose Deprivation-Induced Increases in Protein O-GlcNAcylation and ER Stress

**DOI:** 10.3389/fmolb.2021.780865

**Published:** 2021-12-07

**Authors:** Luyun Zou, Helen E. Collins, Martin E. Young, Jianhua Zhang, Adam R. Wende, Victor M. Darley-Usmar, John C. Chatham

**Affiliations:** ^1^ Division of Molecular and Cellular Pathology, Department of Pathology, University of Alabama at Birmingham, Birmingham, AL, United States; ^2^ Division of Cardiovascular Diseases, Department of Medicine, University of Alabama at Birmingham, Birmingham, AL, United States; ^3^ Birmingham VA Medical Center, Birmingham, AL, United States

**Keywords:** NAD^+^, O-GlcNAc, glucose deprivation, ER stress, calcium, TRPM2 cation channel

## Abstract

The modification of proteins by O-linked β-*N*-acetylglucosamine (O-GlcNAc) is associated with the regulation of numerous cellular processes. Despite the importance of O-GlcNAc in mediating cellular function our understanding of the mechanisms that regulate O-GlcNAc levels is limited. One factor known to regulate protein O-GlcNAc levels is nutrient availability; however, the fact that nutrient deficient states such as ischemia increase O-GlcNAc levels suggests that other factors also contribute to regulating O-GlcNAc levels. We have previously reported that in unstressed cardiomyocytes exogenous NAD^+^ resulted in a time and dose dependent decrease in O-GlcNAc levels. Therefore, we postulated that NAD^+^ and cellular O-GlcNAc levels may be coordinately regulated. Using glucose deprivation as a model system in an immortalized human ventricular cell line, we examined the influence of extracellular NAD^+^ on cellular O-GlcNAc levels and ER stress in the presence and absence of glucose. We found that NAD^+^ completely blocked the increase in O-GlcNAc induced by glucose deprivation and suppressed the activation of ER stress. The NAD^+^ metabolite cyclic ADP-ribose (cADPR) had similar effects on O-GlcNAc and ER stress suggesting a common underlying mechanism. cADPR is a ryanodine receptor (RyR) agonist and like caffeine, which also activates the RyR, both mimicked the effects of NAD^+^. SERCA inhibition, which also reduces ER/SR Ca^2+^ levels had similar effects to both NAD^+^ and cADPR on O-GlcNAc and ER stress responses to glucose deprivation. The observation that NAD^+^, cADPR, and caffeine all attenuated the increase in O-GlcNAc and ER stress in response to glucose deprivation, suggests a potential common mechanism, linked to ER/SR Ca^2+^ levels, underlying their activation. Moreover, we showed that TRPM2, a plasma membrane cation channel was necessary for the cellular responses to glucose deprivation. Collectively, these findings support a novel Ca^2+^-dependent mechanism underlying glucose deprivation induced increase in O-GlcNAc and ER stress.

## Introduction

The posttranslational modification of serine and threonine residues of proteins by O-linked β-N-acetylglucosamine (O-GlcNAc) contributes to the regulation of diverse cellular functions, including epigenetics, Ca^2+^ signaling, metabolism, mitochondrial function, autophagy, and cell survival (Chatham et al., 2021). Sustained increases in cardiac O-GlcNAc levels observed in diabetes and hypertrophy are linked to cardiac dysfunction (Marsh et al., 2014; Mailleux et al., 2016); moreover, increasing evidence suggests that a sustained increase in cardiomyocyte O-GlcNAc levels is sufficient to lead to adverse cardiac remodeling (Prakoso et al., 2021; Umapathi et al., 2021). On the other hand, acute activation of O-GlcNAc levels has been shown to be cardioprotective, whereas loss of O-GlcNAc increases susceptibility to oxidative stress and is associated with increased injury (Ngoh et al., 2010; Wright et al., 2017). Surprisingly, however, despite the important role of O-GlcNAcylation in mediating cardiomyocyte (patho) physiology, our knowledge of the regulation of O-GlcNAcylation pathway remains remarkably limited.

One factor that is widely considered to regulate protein O-GlcNAc levels is nutrient availability (Hart, 2019). However, we have shown that in the perfused rat heart during global ischemia there is a significant increase in cardiac O-GlcNAc levels (Fulop et al., 2007), whereas upon reperfusion nuclear and cytosolic O-GlcNAc levels decline by ∼50% compared to normoxic controls (Laczy et al., 2010). This is an interesting paradox given that the increase in O-GlcNAc is seen during global ischemia, a nutrient deficient state, and the decrease on reperfusion occurs when nutrient availability is no longer limited. This suggests that factors other than nutrient availability also contribute to regulating O-GlcNAc levels. Ischemia also leads to a decrease in nicotinamide adenine dinucleotide (NAD^+^) due to the inability to oxidize NADH, which is one of the many factors contributing to ischemic injury (Matasic et al., 2018). Rather than restore NAD^+^ levels, reperfusion can contribute to further reductions *via* depletion of nicotinamide phosphoribosyltransferase (NAMPT) (Matasic et al., 2018) the rate limiting enzyme in NAD^+^ salvage pathway, as well as activation of poly (ADP-ribose polymerase (PARP) (Szabo et al., 2004), which uses NAD^+^ in the polyADP-ribosylation of proteins. Preserving NAD^+^ levels, either through inhibition of PARP or supplementation with NAD^+^ and its precursors has been shown to decrease injury and improve recovery following ischemia/reperfusion (Szabo et al., 2004; Matasic et al., 2018). Since, maintaining O-GlcNAc levels during reperfusion either by supplementing O-GlcNAc precursors or inhibiting its degradation also improves cardiac function following ischemia reperfusion (Fulop et al., 2007; Laczy et al., 2010), we hypothesized that NAD^+^ and cellular O-GlcNAc levels could be coordinately regulated.

The endoplasmic reticulum (ER) has diverse cellular functions including regulating Ca^2+^ homeostasis, control of lipid and glucose metabolism, and perhaps it is most widely known its role in regulating protein folding and protein quality control (Almanza et al., 2019). When cells are subjected to various stresses which result in Ca^2+^ dysregulation such as nutrient depletion and ischemia/reperfusion, there is impaired protein processing and misfolding of proteins. This triggers a response commonly known as ER stress, which initiates a complex series of signaling events, including the release of Bip/GRP78 from ER stress sensors such as PERK, leading to its phosphorylation (Almanza et al., 2019). There is a subsequent activation of numerous transcriptional events designed to restore ER homeostasis, or if this is not possible lead to the upregulation of proteins such as C/EBP homologous protein (CHOP), which helps regulate ER stress induced apoptosis (Almanza et al., 2019). Interestingly, increasing O-GlcNAc levels has been shown to attenuate ER stress including reducing levels of CHOP (Ngoh et al., 2009) and O-GlcNAcylation of key regulatory proteins such as eukaryotic translation initiation factor 2α (eIF2α) (Jang et al., 2015) appears to play a key role in maintaining ER homeostasis. Raising the possibility that O-GlcNAc mediated regulation of ER stress could be one factor related to the cardioprotective effect of increasing O-GlcNAc. Interestingly, both nutrient deficient states and ischemia/reperfusion, which activate ER stress, also reduce NAD^+^ levels; however, whether NAD^+^ itself to directly regulate ER stress, has not determined.

In support of potential co-regulation of NAD^+^ and O-GlcNAc we have previously reported that exogenous NAD^+^ decreased O-GlcNAc levels in cultured neonatal cardiomyocytes in a time- and dose-dependent manner (Durgan et al., 2011); however, this was under normal unstressed conditions. How NAD^+^ might influence O-GlcNAc levels under cellular stress conditions remains unknown. We have previously reported that glucose deprivation is a potent stimulus for increasing cellular O-GlcNAc levels, which was dependent on extracellular Ca^2+^(Zou et al., 2012). Therefore, using glucose deprivation as a model system in an immortalized human ventricular cell line, we examined the influence of extracellular NAD^+^ on cellular O-GlcNAc levels and ER stress in the presence and absence of glucose. We found that NAD^+^ completely blocked the increase in O-GlcNAc induced by glucose deprivation as well as suppressed the activation of ER stress. We also found that the NAD^+^ metabolites cyclic ADP-ribose (cADPR) and ADPR which are ryanodine receptor (RyR) agonists and caffeine, which activates the RyR, mimicked the effects of NAD^+^. Inhibition of SERCA, which like cADPR, ADPR and caffeine reduce ER/SR Ca^2+^ levels had similar effects on O-GlcNAc and ER stress responses to glucose deprivation. Inhibitors of CaMKII and the Ca^2+^-dependent phosphatase, calcineurin all attenuated the glucose deprivation induced activation of O-GlcNAc and ER stress. Collectively these results suggest novel Ca^2+^-dependent pathway(s) underlying the glucose deprivation induced activation of protein O-GlcNAcylation and ER stress and the disruption of these responses by NAD^+^ and its metabolites.

## Methods

### Antibodies and Reagents

The following primary antibodies were used: anti-O-GlcNAc (CTD110.6 antibody, UAB Epitope Recognition and Immunoreagent Core), anti-GAPDH (Abcam, ab8245), anti-OGA (Santa Cruz, 376429), anti-phospho-PERK (Thr981) antibody (Santa Cruz, sc-32577), and anti-OGT (Sigma, O-6264). Anti-PERK (5683), BiP (3177), CHOP (2895), and acetylated-lysine (9441) were all obtained from Cell Signaling. Anti-pan-ADP-ribose binding reagent was from Millipore (MABE1016). The following secondary antibodies were used: horseradish peroxidase-conjugated anti-mouse IgM (Calbiochem, 401225), anti-mouse IgG (Bio-Rad, 170-6516), and anti-rabbit IgG (Bio-Rad, 170-6515).

The following reagents were obtained from Sigma-Aldrich: β-Nicotinamide adenine dinucleotide hydrate (NAD^+^, N6522), Nicotinamide (NAM, 72340), Sirt1 inhibitor (EX-527, E7034), cyclic adenosine diphosphate ribose (cADPR, C7344), Adenosine 5′-diphosphoribose sodium salt (ADPR, A0752), SERCA inhibitor (cyclopiazonic acid (CPA), C1530), caffeine (C0750), PARP1 inhibitors 3-Aminobenzamide (3AB, A0788) and DR2313 (SML0397), TRPM2 inhibitor [flufenamic acid (FLA), F9005], and the store operated channel inhibitor, (SKF96365, S7809). Glucosamine hydrochloride was obtained from Fluka (49130); note this product has been discontinued and replaced by G4875 from Sigma-Aldrich. The SERCA inhibitor, thapsigargin was obtained from Invitrogen (T7459). The CAMKII inhibitor KN93 (422708) was obtained from Calbiochem. The TRPM2 inhibitor N-(p-Amylcinnamoyl) anthranilic acid (ACA, BML-EI178-0050) was obtained from Enzo. The calcineurin inhibitor, CN585 was obtained from Millipore (207008). The doses of the compounds used in these studies were chosen either based on past studies (Durgan et al., 2011; Zou et al., 2012) or following preliminary dose response curves prior to these studies.

The following cell culture reagents were used: Dulbecco’s modified Eagle’s medium with 1 g/L glucose (Mediateck, Inc.), Dulbecco’s modified Eagle’s medium, no glucose (Gibco), fetal bovine serum (Atlanta Biologicals), and antibiotic-antimycotic (Invitrogen).

### Cell Culture

AC16 cells, originally derived from primary cultures of adult human ventricular heart tissue (Davidson et al., 2005), were used in all studies except where stated otherwise. The recommended media for AC16 cell culture is DMEM/F-12 (Gibco), which includes a 17.5 mM glucose concentration for optimal growth conditions; however, preliminary studies demonstrated that the responsiveness of O-GlcNAc levels to different interventions were substantially blunted at that concentration of glucose. Therefore, we currently use regular DMEM medium, with 5 mM glucose, for culturing AC16 cells and have observed no adverse effects. A limited number of studies used wild type (WT) and TRPM2^−/−^ mouse embryonic fibroblasts, which were cultured under the same conditions as AC16 cells.

At the beginning of the experiment the cell culture media is changed to either fresh regular or glucose-free DMEM, immediately followed by addition of interventions described below. Unless stated otherwise the treatment period is 24 h. The composition of the glucose-free DMEM is identical to regular DMEM except for the lack of both glucose and sodium pyruvate. In an earlier study, we reported that the addition of pyruvate to the glucose free DMEM did not prevent the increase in O-GlcNAc levels (Zou et al., 2012).

At the end of the experiments, all cells were harvested with lysis buffer (20 mM HEPES, 1.5 mM MgCl2, 20 mM KCl, 20% glycerol, 0.2 mM EGTA, 1% Triton X-100, 2 mM Na3VO4, 10 mM NaF, and 2% protease inhibitor, pH 7.9), and kept at –80°C until subsequent analyses.

### Western Blotting

Protein concentrations were determined, and lysates were reduced in 6X sample loading buffer (0.5 M Tris-HCl PH 6.8, 10% SDS, 30% glycerol, 0.2% 2-mercaptoethanol, 0.012% bromophenol blue), boiled for 5 min, separated by SDS PAGE (15 µg of protein/lane), and transferred to Immobilon-P (Millipore) PVDF membrane. Immunoblotting was performed using a rapid immunodetection method for Immobilon-P (Millipore Technical Note TN051). Briefly, the membranes were equilibrated in methanol and air-dried. The dry membrane was incubated in anti-O-GlcNAc antibody CTD110.6 in 1% casein/phosphate-buffered saline (PBS) overnight at 4°C and then washed three times in PBS, as previously described (Zou et al., 2012). Other membranes were equilibrated in PBS; incubated in 5% milk with Tris-buffered saline with 0.01% Tween 20 (TBST) for 1 h for blocking; washed three times in TBST and then incubated with appropriate antibodies in 5% milk/TBST overnight at 4°C and then washed three times in TBST. The membrane was incubated with the appropriate horseradish peroxidase-conjugated secondary antibodies for 1 h at room temperature. After further washing in PBS or TBST the immunoblots were developed with enhanced chemiluminescence (PerkinElmer Life Sciences) using either using either autoradiograph film or digitally using the Amersham Imager 600.

### Statistical Analysis

NIH Image J was used for measuring and analysis of immunoblot densitometry. All data are expressed as mean ± S.E.M. of 3–6 independent experiments and compared by one-way ANOVA follow by Tukey’s test or Student’s *t*-test as appropriate. Statistically significant differences between groups were defined as *p* ≤ 0.05.

## Results

**FIGURE 1:**
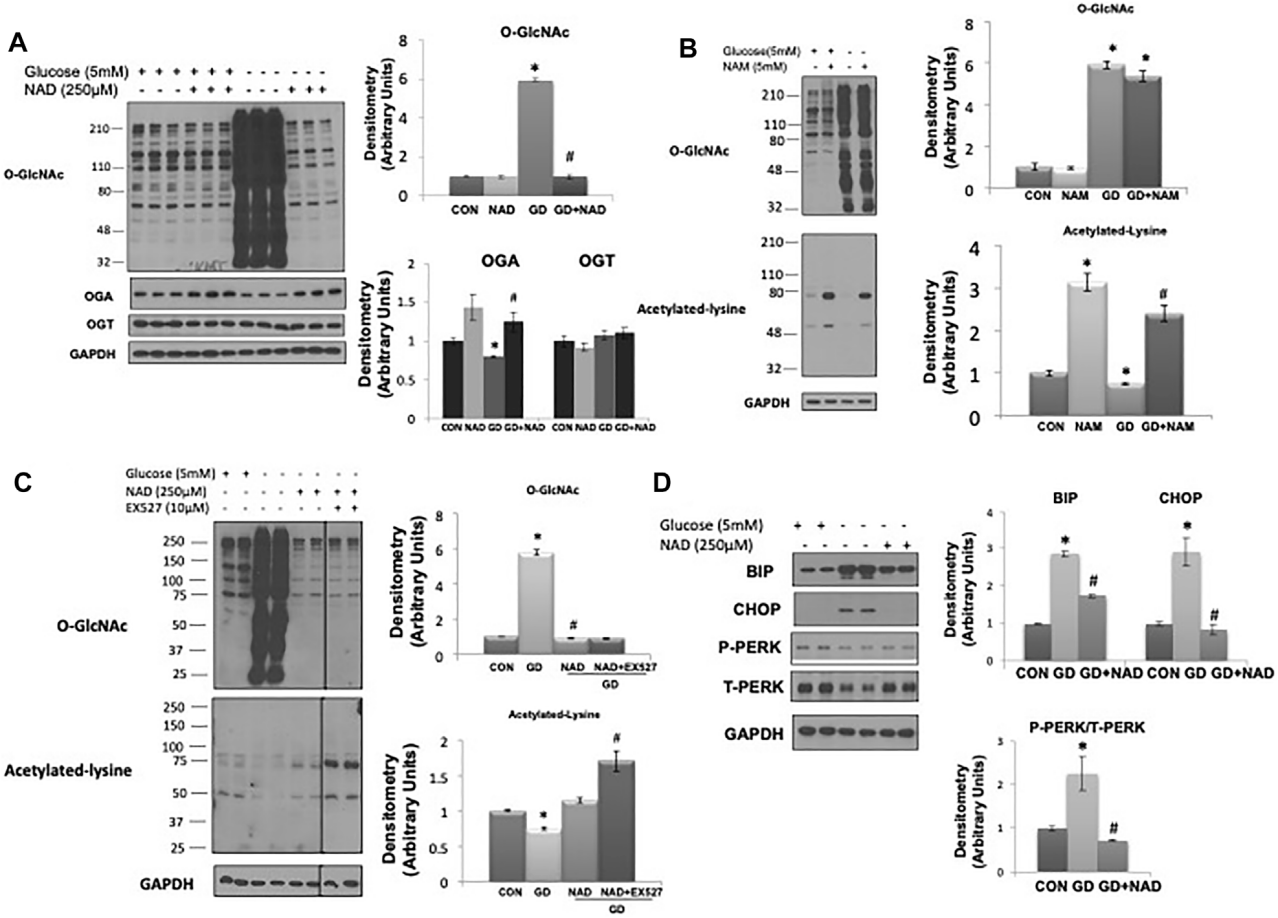
Effects of NAD^+^ on O-GlcNAc levels and ER stress in response to glucose deprivation in AC16 cells. **(A)** Left panel: O-GlcNAc, OGA and OGT immunoblots, with and without glucose (5 mM) and NAD^+^ (250 µM). Right panel: Quantification of immunoblots normalized to GAPDH. **(B)** Left panel: O-GlcNAc and acetylated lysine immunoblots, with and without glucose (5 mM) and nicotinamide (NAM, 5 mM). Right panel: Quantification of immunoblots normalized to GAPDH. **(C)** Left panel: Effects of SIRT1 inhibitor EX527 (10 µM) on O-GlcNAc and acetylated lysine levels in response to glucose deprivation with NAD^+^. Right panel: Quantification of immunoblots normalized to GAPDH. **(D)** Left panel: BiP, CHOP, phospho- (P) and total (T) PERK immunoblots, with and without glucose (5 mM) and NAD^+^ (250 µM). Right panel: Quantification of immunoblots normalized to GAPDH. *p < 0.05 vs. Control (Con) group; #*p* < 0.05 vs. glucose deprivation (GD) group. All data are expressed as mean ± S.E.M. of 3–6 independent experiments.

Consistent with our previous report (Zou et al., 2012) glucose deprivation resulted in a robust increase in O-GlcNAc levels (Figure 1A). OGA protein levels decreased with glucose deprivation and OGT levels were unchanged as previously reported (Zou et al., 2012). Surprisingly, NAD^+^ (250 µM) completely blocked the increase in O-GlcNAc (Figure 1A). NAM, a cell membrane permeable metabolite of NAD^+^ had no effect on basal O-GlcNAc levels and did not block the increase in O-GlcNAc in response to glucose deprivation (Figure 1B). NAM increased protein acetylation levels under basal and glucose deprivation conditions (Figure 1B), consistent with its effect as a pan-sirtuin (SIRT) inhibitor (Bheda et al., 2016; Jiang et al., 2017). To determine if there was a role for SIRTs in mediating the effects of NAD^+^, we examined whether EX527, a SIRT1 and to a lesser extent SIRT2 inhibitor (Peck et al., 2010), altered the response of O-GlcNAc to NAD^+^. EX527, had no effect on the suppression of O-GlcNAc levels by NAD^+^ in response to glucose deprivation; although there was an increase in protein acetylation levels, consistent with its action as a SIRT inhibitor (Figure 1C).

It had been reported that increased CTD110.6 immunoreactive bands due to glucose deprivation were a result of cross-reactivity with the attenuated *N*-linked glycan, chitobiose Asn-GlcNAc-GlcNAc (Isono, 2011); however, our earlier study clearly demonstrated that the increase CTD110.6 positive bands in response to glucose deprivation was predominantly due to increased O-GlcNAc levels (Zou et al., 2012).

**FIGURE 2:**
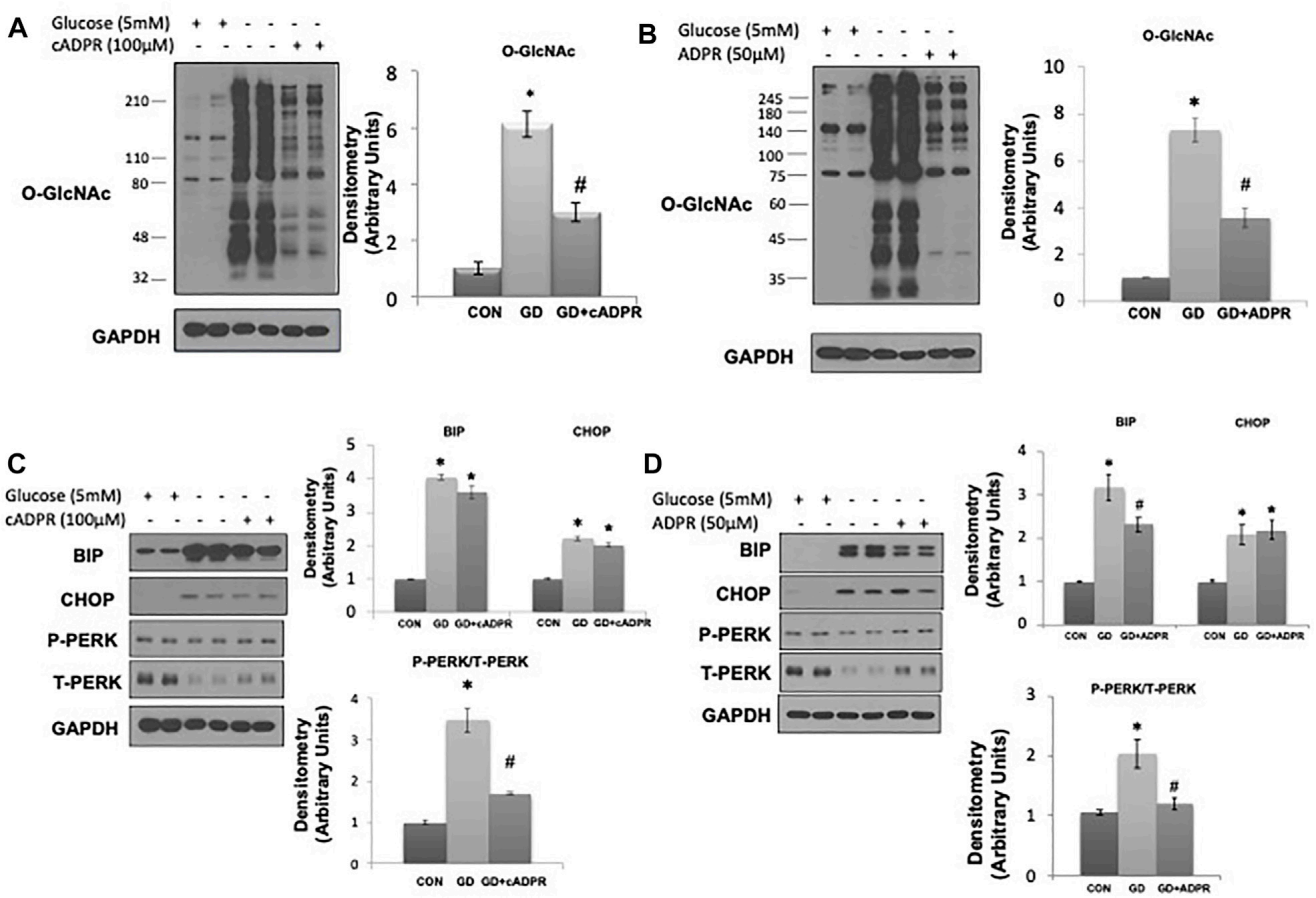
The effects of cADPR and ADPR on the glucose deprivation induced increase in O-GlcNAc and ER Stress in AC16 cells. **(A)** Left panel: O-GlcNAc immunoblots with and without glucose in the presence or absence of cADPR (100 µM). Right panel Quantification of immunoblots normalized to GAPDH. **(B)** Left panel: O-GlcNAc immunoblots with and without glucose in the presence or absence of ADPR (50 µM). Right panel: Quantification of immunoblots normalized to GAPDH. **(C)** Left panel: BiP, CHOP, phospho- (P) and total (T) PERK immunoblots, with and without glucose in the presence or absence of cADPR (100 µM). Right panel: Quantification of immunoblots normalized to GAPDH. **(D)** Left panel: BiP, CHOP, phospho- (P) and total (T) PERK immunoblots, with and without glucose in the presence or absence of ADPR (50 µM). Right panel: Quantification of immunoblots normalized to GAPDH. *p < 0.05 vs. Control (Con) group; #*p* < 0.05 vs. glucose deprivation (GD) group. All data are expressed as mean ± S.E.M. of 3–6 independent experiments.

As glucose deprivation is known to induce ER stress (Malhotra and Kaufman, 2007;De la Cadena et al., 2014), we examined whether NAD^+^ also blunted this response. Glucose deprivation increased levels of the ER stress response elements BiP/GRP78, phospho-to-total PERK and CHOP (Figure 1D), all of which were attenuated by NAD^+^. The NAD^+^ metabolite, NAM, had no effect on the O-GlcNAc response to glucose deprivation; however, NAD^+^ can also be metabolized to cADPR and ADPR (Malavasi et al., 2008; Ernst et al., 2013). We found that both cADPR and ADPR blocked the glucose-deprivation induced increase in O-GlcNAc levels (Figures 2A,B) and like NAD^+^ they also blocked the glucose deprivation induced increase in phospho-to-total PERK, but they had minor effects on the increases in BiP/GRP78 and CHOP (Figures 2C,D).

**FIGURE 3:**
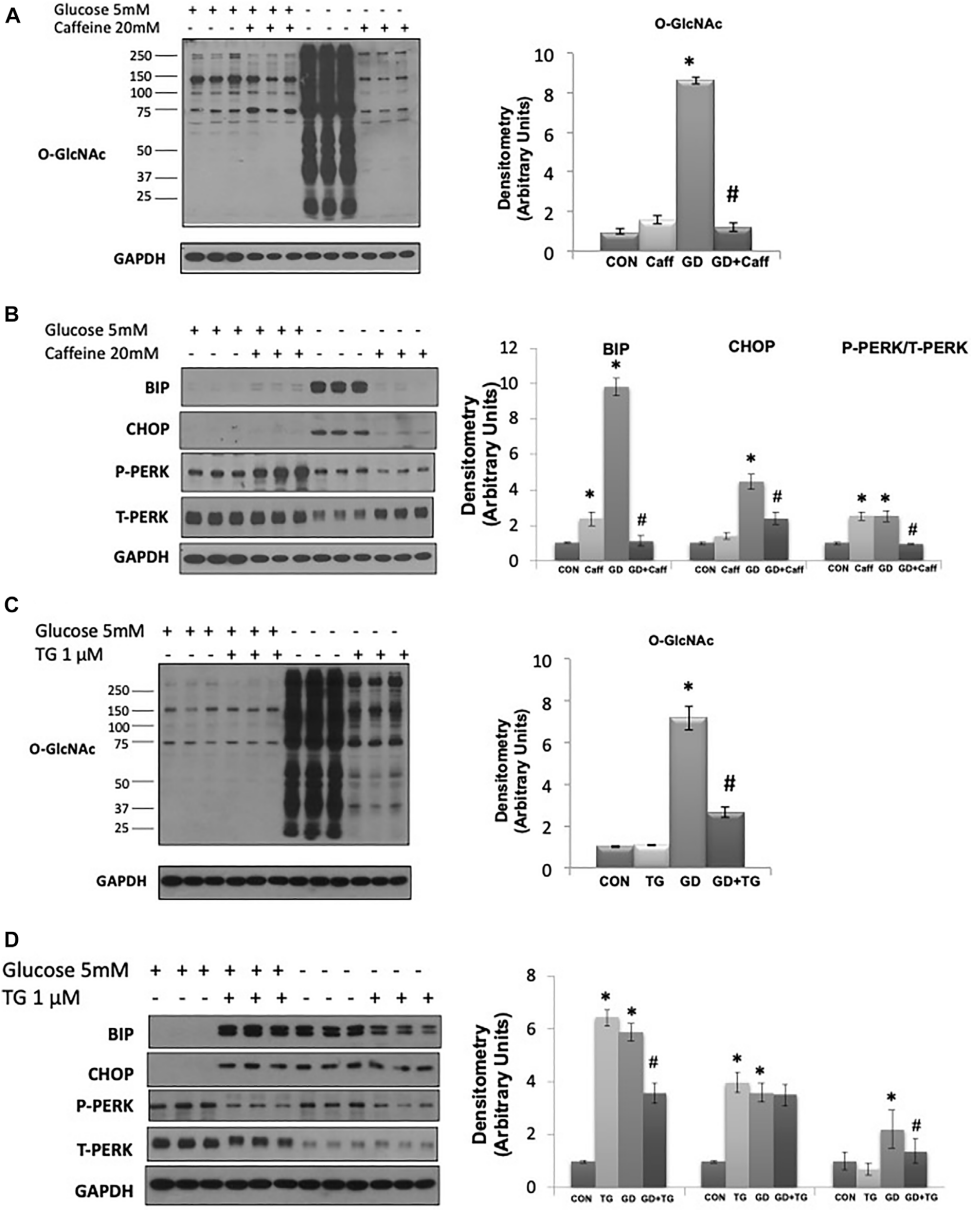
The effects of caffeine and thapsigargin (TG) on the glucose deprivation induced increase in O-GlcNAc and ER Stress in AC16 cells: **(A)** Left panel: O-GlcNAc immunoblots with and without glucose in the presence or absence of caffeine (5 mM). Right panel: Quantification of immunoblots normalized to GAPDH. **(B)** Left panel: BiP, CHOP, phospho- (P) and total (T) PERK immunoblots, with and without glucose in the presence or absence of caffeine (5 mM). Right panel: Quantification of immunoblots normalized to GAPDH. **(C)** Left panel: O-GlcNAc immunoblots with and without glucose in the presence or absence of thapsigargin (1 µM). Right panel Quantification of immunoblots normalized to GAPDH. **(D)** Left panel: BiP, CHOP, phospho- (P) and total (T) PERK immunoblots, with and without glucose in the presence of thapsigargin (1 µM). Right panel: Quantification of immunoblots normalized to GAPDH. All data are expressed as mean ± S.E.M. of 3–6 independent experiments. *p < 0.05 vs. Control (Con) group; #*p* < 0.05 vs. glucose deprivation (GD) group.

A potential intracellular target of both cADPR and ADPR is the RyR, resulting in ER/SR Ca^2+^ release (Bastide et al., 2002;Lee, 2012;Ernst et al., 2013;Guse, 2015). We found that caffeine, a widely used RyR agonist, also blocked the glucose deprivation induced increase in O-GlcNAc (Figure 3A). Due to its effects on ER/SR Ca^2+^ homeostasis caffeine alone resulted in modest increases in BiP/GRP78 and phospho-to-total PERK; however, under conditions of glucose deprivation caffeine treatment blocked the increases in BiP/GRP78, CHOP, and phospho-to-total PERK (Figure 3B). To determine if these results were specific for activation of the RyR or whether a reduction in ER/SR Ca^2+^ levels in general were sufficient, we examined the effects of the SERCA inhibitor thapsigargin, which also decreases ER/SR Ca^2+^ levels (Krebs et al., 2015). We found that thapsigargin significantly attenuated the glucose deprivation-induced increase in O-GlcNAc (Figure 3C). As expected thapsigargin alone induced ER stress resulting in increases in BiP/GRP78 and CHOP in the presence of glucose; however, thapsigargin attenuated the glucose deprivation-induced increase in BiP/GRP78 and phospho-to-total PERK (Figure 3D). Cyclopiazonic acid (CPA) another SERCA inhibitor had similar effects to thapsigargin on the O-GlcNAc and ER stress responses to glucose deprivation (Supplementary Figure S1). These data are consistent with a mechanism by which cADPR and ADPR attenuate the cellular response to glucose deprivation *via* activation of the RyR and lowering ER/SR Ca^2+^ levels.

**FIGURE 4:**
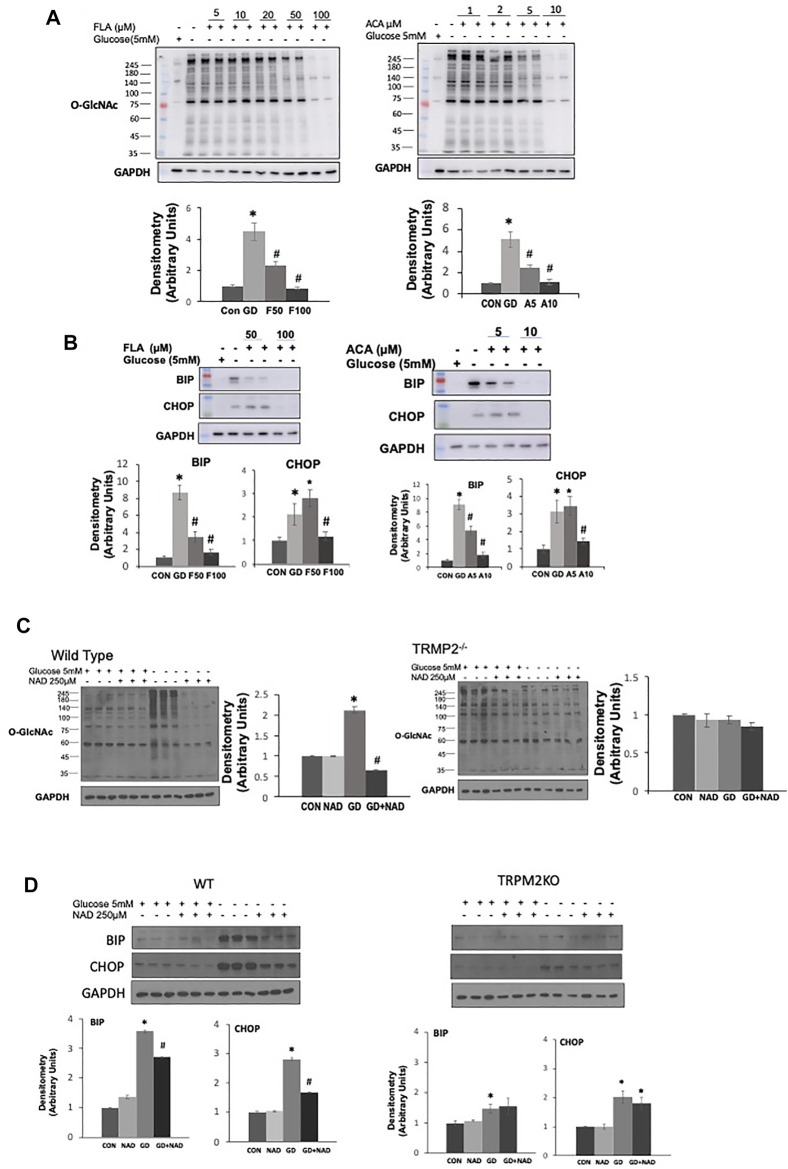
The effects of TRPM2 inhibition in AC16 cells, or TRPM2 deletion in MEFs on the glucose deprivation induced increase in O-GlcNAc and ER Stress: **(A)** Top Panels: O-GlcNAc immunoblots with and without glucose in the presence or absence of TRPM2 inhibitors FLA (5–100 µM) and ACA (1–10 µM); Bottom Panels: Quantification of immunoblots at 50 and 100 µM FLA and 5 and 10 µM ACA normalized to GAPDH. **(B)** Top Panels: BiP and CHOP immunoblots with and without glucose in the presence or absence of TRPM2 inhibitors FLA (50, 100 µM) and ACA (5, 10 µM); Bottom Panels: Quantification of immunoblots normalized to GAPDH. **(C)** Left O-GlcNAc immunoblots from wild type (WT) and TRPM2^−/−^ cells with and without glucose in the presence or absence of NAD^+^ (250 µM); Quantification of immunoblots for WT and TRMP2^−/−^ normalized to GAPDH. **(D)** Top Panels: Immunoblots for BiP and CHOP from wild type (WT) and TRPM2^−/−^ cells with and without glucose in the presence or absence of NAD^+^ (250 µM); Bottom Panels: Quantification of immunoblots for WT and TRPM2^−/−^ normalized to GAPDH. *p < 0.05 vs. Control (Con) group; #*p* < 0.05 vs. glucose deprivation (GD) group. All data are expressed as mean ± S.E.M. of 3–6 independent experiments.

To try and determine whether the effects of ADPR might be mediated *via* a different mechanism than cADPR, we examined whether inhibiting poly-ADPR polymerase (PARP) enzymes, which are responsible for catalyzing poly-ADP ribosylation protein modification (Hottiger, 2015) prevented the effects of NAD^+^ on the response to glucose deprivation. We used two PARP inhibitors 3AB and DR2313 and found that neither inhibitor blocked the effects of NAD^+^ in preventing the increase in O-GlcNAc; however, they both reduced overall protein ADP-ribosylation levels at the same concentrations, demonstrating their effectiveness at inhibiting PARP under the same conditions (Supplementary Figure S2). The transient receptor potential melastatin 2 (TRPM2) protein, a Ca^2+^ permeable, non-selective cation channel is activated by ADPR binding to the C-terminal domain (Sumoza-Toledo and Penner, 2011; Ernst et al., 2013). cADPR has also been reported to activate TRPM2 (Sumoza-Toledo and Penner, 2011). Therefore, we speculated that TRPM2 inhibition might prevent cADPR/ADPR from blocking the glucose deprivation induced increase in O-GlcNAc. Surprisingly however, two different TRPM2 inhibitors, FLA and ACA (Kraft et al., 2006; Naziroglu et al., 2007), attenuated the glucose deprivation induced increase in O-GlcNAc in a concentration-dependent manner (Figure 4A). Consistent with other interventions that attenuated the increase in O-GlcNAc they both attenuated the activation of the ER stress response as indicated by lower levels of BiP/GRP78 and CHOP (Figure 4B). To further explore the role of TRPM2 in mediating the response to glucose deprivation and rule out potential off target effects of FLA and ACA, we examined the effects of NAD^+^ on O-GlcNAc levels in wild type and TRPM2^−/−^ mouse embryonic fibroblasts (MEFs). In wild type cells we observed the expected increase in O-GlcNAc levels in response to glucose deprivation, which was suppressed by NAD^+^; however, in TRPM2^−/−^ cells glucose deprivation did not result in an increase in O-GlcNAc levels, supporting the findings with TRPM2 inhibitors (Figure 4C). NAD^+^ had no effect on O-GlcNAc levels in TRPM2^−/−^ cells regardless of the presence or absence of glucose. Following glucose deprivation, WT MEFs exhibited >3-fold increase in BiP/GRP78 and CHOP levels, which was attenuated by NAD^+^ (Figure 4D), consistent with the responses of AC16 cells (Figure 1D). However, TRPM2^−/−^ cells exhibited very modest increases in BiP/GRP78 and CHOP in response to glucose deprivation and these changes were unaffected by the presence of NAD^+^ (Figure 4D). These findings suggest that the effects of cADPR/ADPR are not mediated by activation of TRPM2, but instead revealed that TRPM2 is a potential a mediator of the cellular responses to glucose deprivation.

**FIGURE 5:**
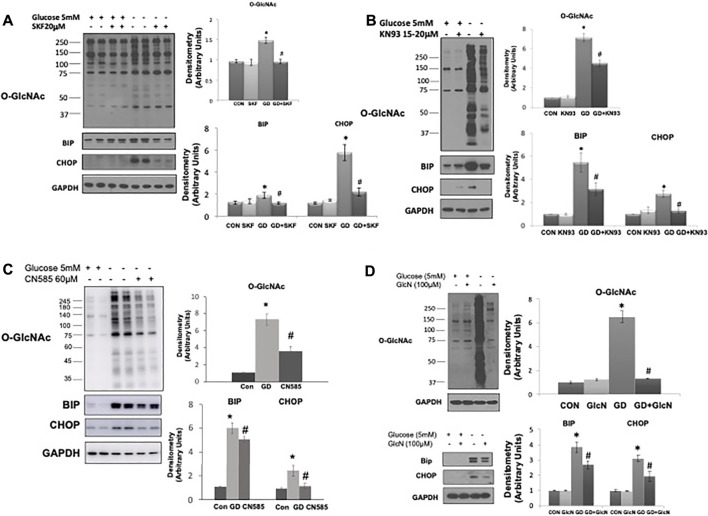
The effects of SKF96365, KN93, CN585, and glucosamine on the glucose deprivation induced increase in O-GlcNAc levels and indices of ER stress in AC16 cells. **(A)** Left Panel: Immunoblots O-GlcNAc, BiP and CHOP with and without glucose in the presence or absence of SKF96365 (20 µM); Right Panels: Quantification of immunoblots normalized to GAPDH. (Note the duration of these experiments was shortened from 24 to 6 h because longer periods of glucose deprivation in the presence of SKF96365 resulted in cell death; hence the increase in O-GlcNAc following glucose deprivation is lower than other studies where the experimental period was 24 h), **(B)** Left Panels: Immunoblots for O-GlcNAc, BiP and CHOP with and without glucose in the presence or absence of KN93 (20 µM); Right Panels: Quantification of immunoblots normalized to GAPDH. **(C)** Left Panels: Immunoblots for O-GlcNAc, BiP and CHOP with and without glucose in the presence or absence of CN585 (60 µM); Right Panels: Quantification of immunoblots normalized to GAPDH. **(D)** Left Panel: Immunoblots O-GlcNAc, BiP and CHOP with and without glucose in the presence or absence of glucosamine; Right Panels: Quantification of immunoblots normalized to GAPDH. *p < 0.05 vs. Control (Con) group; #*p* < 0.05 vs. glucose deprivation (GD) group. All data are expressed as mean ± S.E.M. of 3–6 independent experiments.

The observation that TRPM2, a Ca^2+^ permeable cation channel, appears to be necessary for glucose deprivation induced activation of O-GlcNAc is consistent with our earlier study indicating that influx of extracellular Ca^2+^ was needed for glucose deprivation-induced activation of O-GlcNAc levels in cardiomyocytes (Zou et al., 2012). However, in those studies we did not determine ER stress responses. Here we found that as previously reported (Zou et al., 2012), the store operated Ca^2+^ channel inhibitor SKF9635 blunted the increase in O-GlcNAc; importantly however, it also attenuated the glucose deprivation induced increase in BiP/GRP78 and CHOP (Figure 5A). We also found that as previously shown (Zou et al., 2012) the CaMKII inhibitor KN93 attenuated the glucose deprivation induced increase in O-GlcNAc, it also blunted the increase in BiP/GRP78 and CHOP (Figure 5B). Recent studies have suggested that KN93 directly binds Ca^2+^/CaM (Pellicena and Schulman, 2014; Wong et al., 2019), thereby, potentially influencing other Ca^2+^/CaM protein interactions. The Ca^2+^-dependent protein phosphatase, calcineurin is a well-established target for Ca^2+^/CaM and has been associated with the regulation of ER stress (Carreras-Sureda et al., 2018). We found that CN585 a highly selective calcineurin inhibitor (Erdmann et al., 2010), blunted the glucose deprivation induced increases in O-GlcNAc, BiP/GRP78, and CHOP (Figure 5C).

In our earlier study we found that low concentrations of glucosamine, attenuated the increase in O-GlcNAc resulting from glucose deprivation (Zou et al., 2012). Here we show that not only does glucosamine block the increase in O-GlcNAc levels, but it also blunted the increase in BiP/GRP78 and CHOP that occurs in response to glucose deprivation (Figure 5D). Thus, all interventions tested thus far, that attenuate the glucose deprivation induced increase in O-GlcNAc, also attenuate the ER stress response, consistent with a common underlying activation mechanism.

## Discussion

The primary regulation of cellular O-GlcNAc levels is commonly linked to glucose availability, with increased glucose levels leading to higher O-GlcNAc levels and vice versa. However, there are reasons to believe that other factors could be important in the short-term regulation of O-GlcNAc levels. Of particular relevance are the number of studies that have shown that glucose deprivation is a potent stimulus for increasing O-GlcNAc levels (Cheung and Hart, 2008; Taylor et al., 2008; Taylor et al., 2009; Zou et al., 2012). Interestingly glucose deprivation is also known to lead to a decrease in NAD^+^ levels and we have previously shown that exogenous NAD^+^ decreased cardiomyocyte O-GlcNAc levels without changes in substrate availability (Durgan et al., 2011). We proposed therefore that cellular NAD^+^ and O-GlcNAc levels may be coordinately regulated. Using glucose deprivation in an immortalized human ventricular cell line (AC16 cells) as a model system, we found that NAD^+^ and its metabolites cADPR and ADPR, completely blocked the increase in O-GlcNAc induced by glucose deprivation while also suppressing activation of ER stress. NAD^+^ is a potential activator of glycolysis thereby possibly increasing pyruvate availability; however, we have shown that the addition of pyruvate had no effect on the response to glucose deprivation (Zou et al., 2012), suggesting that activation of glycolysis was not the mechanism underlying the effects of NAD^+^. cADPR and ADPR are putative ryanodine receptor (RyR) agonists, and caffeine, which also activates the RyR and lowers ER/SR Ca^2+^ levels, mimicked the effects of NAD^+^, cADPR, and ADPR. Other treatments which also reduce ER/SR Ca^2+^ levels, such as SERCA inhibitors thapsigargin and CPA, had similar effects to NAD^+^ on O-GlcNAc and ER stress responses to glucose deprivation (Figures 2, 3, Supplementary Figure S1). Collectively, these observations suggest a potential common mechanism regulating O-GlcNAc levels in response to glucose deprivation. The fact that inhibition or deletion of the plasma membrane cation channel TRPM2 blocked the effects of NAD^+^ and glucose deprivation (Figure 4) suggests that Ca^2+^ signaling may be a common element in both the glucose deprivation induced increase in O-GlcNAc and ER stress and the inhibition of these responses by NAD^+^.

Additional support for a role of Ca^2+^ in mediating the cellular response to glucose deprivation is that KN93, a CaMKII inhibitor, attenuated both the increase in O-GlcNAc and the ER stress response to glucose deprivation, like that seen with NAD^+^ (Figure 5B). KN93, one of the most widely used CaMKII inhibitors, does so by directly binding Ca^2+^/CaM (Pellicena and Schulman, 2014; Wong et al., 2019) rather than CaMKII itself thereby, potentially inhibiting other Ca^2+^/CaM protein interactions. The Ca^2+^-dependent protein phosphatase, calcineurin is a well-established Ca^2+^/CaM target and has been associated with the regulation of ER stress (Carreras-Sureda et al., 2018). There is also evidence, of crosstalk been calcineurin and O-GlcNAc regulation in cardiomyocytes (Facundo et al., 2012). Here we found that CN585, a highly selective calcineurin inhibitor (Erdmann et al., 2010), exhibited similar effects to both KN93 and NAD^+^ by blunting the glucose deprivation induced increases in O-GlcNAc, BiP/GRP78, and CHOP (Figure 5C). Additional studies are needed to determine how Ca^2+^/CaM or calcineurin activation initiates ER stress and increases O-GlcNAc levels; nevertheless, these data suggest than canonical Ca^2+^ signaling pathways contribute to the regulation of O-GlcNAc and ER stress following glucose deprivation.

**FIGURE 6:**
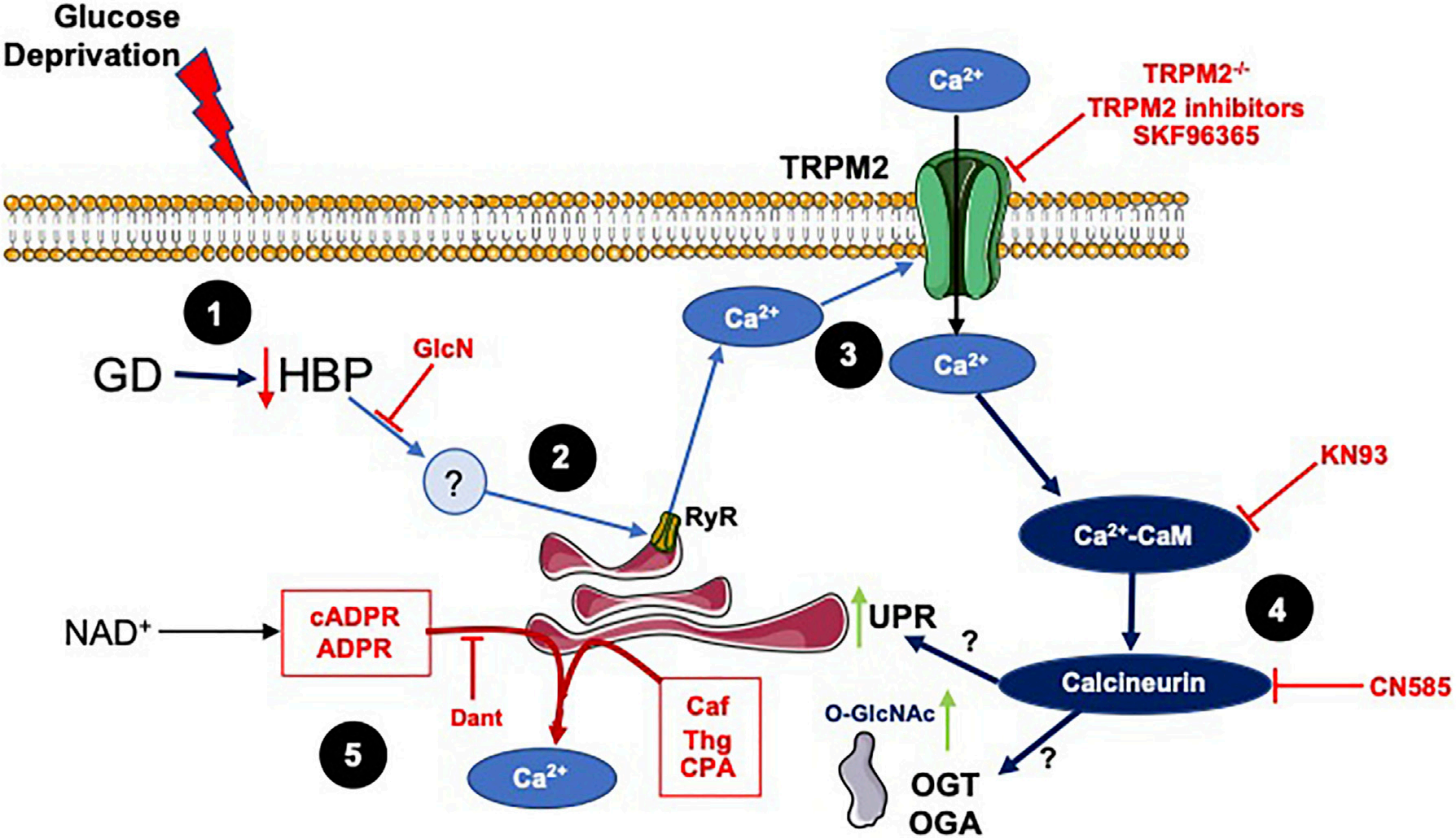
A schematic summarizing the results and illustrating potential mechanisms underlying effects of glucose deprivation on O-GlcNAc and ER stress and how they might be regulated by NAD^+^ and its metabolites: 1) In response to glucose deprivation there is a decrease in HBP flux, which is supported by the observation that glucosamine, which is metabolized to UDP-GlcNAc *via* the HBP, attenuates the responses to glucose deprivation. 2) While the downstream effector of the decreased HBP flux has yet to be identified, a likely candidate would the loss of O-GlcNAc from specific site(s) on a protein(s) subsequently triggering a release of Ca^2+^ from the ER/SR. This is supported by observations that interventions that decrease ER/SR Ca^2+^ levels attenuate the responses to glucose deprivation. 3) Inhibition of TRPM2 or loss of TRPM2 blocked the glucose deprivation responses, suggesting that its activation *via* glucose deprivation resulted in an influx of extracellular Ca^2+^. In addition, ER/SR Ca^2+^ release has been shown to be sufficient to activate TRPM2 channels. This is supported by earlier observations that extracellular Ca^2+^ was required for the glucose deprivation induced increase in O-GlcNAc (Zou et al., 2012) and that inhibition of downstream Ca^2+^ signaling pathways blunted the increase in ER stress and O-GlcNAc in response to glucose deprivation. In addition, SKF96365 a SOCE inhibitor, which also attenuates the response to glucose deprivation, is reported to inhibit members of the transient receptor potential family including TRPM2 channels (Harteneck et al., 2011). 4) The TRPM2 mediated influx of Ca^2+^ activates Ca^2+^/CaM-dependent pathway, which potentially *via* activation of calcineurin leads to increased ER stress and O-GlcNAc levels. This is supported by the observation that KN93 a CaMKII and Ca^2+^/CaM inhibitor and CN585, a calcineurin inhibitor, attenuated the responses to glucose deprivation. The links between Ca^2+^/CaM and the increase in ER stress and O-GlcNAc following glucose deprivation are currently not known, although calcineurin has been associated with activation of ER stress (Carreras-Sureda et al., 2018). 5) The NAD^+^ metabolites, cADPR and ADPR, are known to activate the RyR releasing ER/SR Ca^2+^ and caffeine a RyR agonist all attenuate the cellular responses to glucose deprivation. The SERCA inhibitors thapsigargin (Thg) and CPA, which decrease ER/SR Ca^2+^ levels also blunt the response to glucose deprivation.

Collectively these studies suggest a common pathway for activation of both O-GlcNAc levels and ER stress, in response to glucose deprivation consistent with the coordinated mobilization of intra- and extra-cellular Ca^2+^. This process appears to be interfered with by NAD^+^, thereby, blocking the increase in O-GlcNAc levels and ER stress. Based on these data a schematic outlining potential early cellular responses to glucose deprivation as well as potential mechanism by which NAD^+^ blocks this response is shown in Figure 6. While there could be parallel, independent activation of O-GlcNAc and ER stress, we cannot rule out the possibility that their activation is interdependent. For example, ER stress has been directly linked to the activation of the HBP (Wang et al., 2014) and O-GlcNAc signaling has been shown to influence ER stress (Ngoh et al., 2009).

Consistent with our earlier report (Zou et al., 2012), we found that 100 µM glucosamine blocked the increase in O-GlcNAc; here we show it also attenuated the ER stress response to glucose deprivation (Figure 5D). Glucosamine is phosphorylated by hexokinase to glucosamine-6-phosphate and subsequently to UDP-GlcNAc *via* the HBP. Since increasing the HBP flux with the addition of glucosamine blocks the cellular responses to glucose deprivation, it seems likely that a decrease in HBP flux triggers these responses. This is consistent with other studies indicating glucose deprivation induced increase in O-GlcNAc was associated with decreased HBP flux (Taylor et al., 2009; Zou et al., 2012). Since activation of the HBP attenuates the effects of glucose deprivation, it appears unlikely that OGT activation contributes to the increase in O-GlcNAcylation. Future studies using recently available selective OGT inhibitors (Martin et al., 2018) could be used to examine this question further.

Since NAD^+^ metabolites cADPR and ADPR blocked the effects of glucose deprivation, an outstanding question is whether NAD^+^ can directly cross the plasma membrane. It has been suggested that extracellular NAD^+^ cannot cross the plasma membrane but must undergo extracellular hydrolysis by NAD hydrolases such as CD38 and CD157 producing cADPR and ADPR which are membrane permeable (Malavasi et al., 2008). On the other hand, others have reported that Connexin 43 hemichannels (Cx43) facilitate the bidirectional transport of NAD^+^ (Bruzzone et al., 2001; Wang et al., 2013), thereby, providing a pathway for NAD^+^ to directly enter the cell. We did not measure intracellular NAD^+^ levels in our studies, thus we do not know whether NAD^+^ directly entered the cells. If cADPR and ADPR result from extracellular hydrolysis it is also possible that they could act on the purinergic receptors. Interestingly, ADPR has been shown to specifically activate the purinergic P2Y1 receptor (Gustafsson et al., 2011). Even if the primary mechanism of action of cADPR and ADPR is *via* activation of purinergic receptors a common downstream consequence is the IP_3_R dependent release of ER Ca^2+^ stores (Huang et al., 2021), which would be consistent with the overall concept that mobilization of intracellular Ca^2+^ stores is involved in these responses. However, we found that CD38 null MEFs exhibited the same response to glucose deprivation as WT MEFs and did not blunt the response of NAD^+^ (data not shown). Therefore, at least in these studies CD38 mediated hydrolysis of NAD^+^ did not play a role in its effects on the responses to glucose deprivation; however, this does not rule out possible roles of other extracellular NAD^+^ hydrolases (Malavasi et al., 2008).

Regardless of whether NAD^+^ can directly cross the plasma membrane, cADPR and ADPR are both known to activate RyR (Bastide et al., 2002; Lee, 2012; Ernst et al., 2013; Guse, 2015). Caffeine a known RyR agonist had the same effects as, cADPR and ADPR on the responses to glucose deprivation and SERCA inhibitors thapsigargin and CPA, which also decrease ER/SR Ca^2+^ levels, blunts the responses to glucose deprivation. Collectively these findings suggest that full ER/SR Ca^2+^ stores are required to initiate the cellular responses to glucose deprivation and that mistimed release of these stores *via* RyR activation or SERCA inhibition interferes with the downstream response to glucose deprivation. Additional pharmacological approaches to independently lower or increase cytosolic Ca^2+^ levels would help to further dissect the specific cytosolic pools of Ca^2+^ involved in the response to glucose deprivation. We have shown that extracellular Ca^2+^ was required for the glucose deprivation induced increase in O-GlcNAc suggesting that the influx of extracellular Ca^2+^ is a necessary part of this process (Zou et al., 2012); however, the specific Ca^2+^ entry pathway was not identified.

Unexpectedly, we found that inhibition of the plasma membrane cation channel TRPM2 in AC16 cells and the deletion of TRMP2 in MEFs completely abrogated the responses of O-GlcNAc and ER stress to glucose deprivation in both cell types. The results from this study suggest that TRPM2 is very likely the Ca^2+^ channel that is responsible for influx of extracellular Ca^2+^ following glucose deprivation. Our studies also suggest that the cellular responses to glucose deprivation requires full ER/SR Ca^2+^ stores. One established Ca^2+^ signaling pathway that requires both full ER/SR Ca^2+^ stores and influx of extracellular Ca^2+^ is store operated calcium entry (SOCE), in which a transient release of Ca^2+^ from the ER/SR triggers, in a highly coordinated manner, the influx of extracellular Ca^2+^ *via* plasma membrane cation channels, leading to a more sustained increase in intracellular Ca^2+^ (Collins et al., 2013). TRPM2 is not recognized as a classical SOCE channel; however, both intra- and extracellular Ca^2+^ are required for its full activation (Sumoza-Toledo and Penner, 2011). Moreover, Ca^2+^ release from intracellular stores is both necessary and sufficient for the initial activation of TRPM2 (Du et al., 2009). In addition, SKF96365, a SOCE inhibitor, also inhibits transient receptor potential family including TRPM2 channels (Harteneck et al., 2011); thus, the effect of SKF96365 on attenuating the O-GlcNAc and ER stress response to glucose deprivation (Figure 5A) could be due to inhibition of TRPM2 mediated Ca^2+^ influx. The mechanism by which the influx of Ca^2+^ regulates ER stress and O-GlcNAc levels remains to be determined; however, Ca^2+^/CaM-mediated Ca^2+^ signaling has been shown to regulate ER stress *via* activation of calcineurin (Carreras-Sureda et al., 2018). Further studies are needed to better understand the Ca^2+^-dependent regulation of O-GlcNAcylation.

While we cannot rule out contributions from other plasma membrane cation channels in mediating the responses to glucose deprivation, these data provide strong evidence for a role for TRPM2 in mediating this stress response. TRPM2 is widely expressed (Miller and Cheung, 2016), however, it has not been previously linked to the cellular response to glucose deprivation, ER stress or O-GlcNAcylation. Interestingly, in the heart TRPM2 activation was found to be protective against ischemia/reperfusion injury (Miller et al., 2013; Miller et al., 2014). TRPM2 has also plays a role in mediating oxidative stress induced increase in intracellular Ca^2+^ (Li et al., 2017) and appears to be upstream of several cell death pathways (Shi et al., 2021). It has also been linked to the regulation of insulin signaling and glucose metabolism in a Ca^2+^/calmodulin dependent fashion in the heart (Zhang et al., 2012). Therefore, while unexpected, it is not unrealistic that TRPM2 channels could be involved in mediating cellular stress responses such as glucose deprivation. Future studies using TRPM2-KO mice (Yamamoto et al., 2008) or cell-type specific TRPM2 deletion will provide addition insights into the role of TRPM2 mediated Ca^2+^ signaling and O-GlcNAc regulation, and ER stress activation. It is also possible that other Ca^2+^ channels could contribute to stress responses such as glucose deprivation in a tissue and cell-type dependent manner.

Together these findings indicate that the initial response to glucose deprivation is the coordinated release of ER/SR Ca^2+^, which triggers TRPM2 channel opening, the influx of extracellular Ca^2+^, and the subsequent activation of canonical Ca^2+^/CaM signaling pathways. One possible mechanism by which a glucose deprivation induced decrease in HBP flux could trigger such a response could be the loss of O-GlcNAc on a protein or proteins that regulates ER Ca^2+^ release. While identifying a specific O-GlcNAc target, is beyond the scope of this study, one possibility could be changes in O-GlcNAcylation of the RyR itself, although it remains unclear whether RyR is an O-GlcNAc target (Okolo, 2019; Popescu et al., 2019). Interestingly, TRPM2 has multiple phosphorylation sites (Hornbeck et al., 2015) and overall tyrosine phosphorylation has been shown to regulate TRPM2 activity (Zhang et al., 2007). Thus, it is possible that a decrease in O-GlcNAcylation of specific TRPM2 sites could result in an increase in TRPM2 phosphorylation, thereby enhancing its activation by ER/SR Ca^2+^ release. Conclusively demonstrating that the loss of O-GlcNAc from a specific site on one or more proteins initiates the response to glucose deprivation will be very challenging; however, RyR and TRPM2 are intriguing possibilities.

In conclusion, while nutrient availability is widely considered to be the major regulator of cellular O-GlcNAc levels, there are a number of situations where this does not appear to be the case including ischemia/reperfusion (Fulop et al., 2007; Laczy et al., 2010) and glucose deprivation (Zou et al., 2012). We have previously reported that NAD^+^ decreased O-GlcNAc levels in unstressed cardiomyocytes (Durgan et al., 2011), suggesting the potential for coordinated regulation between O-GlcNAc and NAD^+^. Therefore, using glucose deprivation, a potent activator of O-GlcNAc levels, as a model system we examined how NAD^+^ influenced the response of O-GlcNAc levels and ER stress in the presence and absence of glucose. Our data shown here combined with earlier studies (Zou et al., 2012), suggests that in response to glucose deprivation a decrease in HBP flux leads to a coordinated release of Ca^2+^ from the ER/SR, leading to a subsequent influx of extracellular Ca^2+^ and activation of canonical Ca^2+^/CaM-mediated Ca^2+^ signaling pathway, which activates O-GlcNAc levels and ER stress. NAD^+^ appears to disrupt these responses by the mistimed release of intracellular Ca^2+^ likely *via* RyR activation by its metabolites cADPR and ADPR. We have also identified TRPM2, a widely expressed plasma membrane cation channel (Sumoza-Toledo and Penner, 2011), as a potential key mediator of the cellular responses to glucose deprivation. Nevertheless, detailed characterization of the electrophysiological responses to glucose deprivation are clearly needed to identify the specific source of Ca^2+^ release from the ER/SR as well as the biophysical properties of the Ca^2+^ influx channel(s) involved, and how this is affected by NAD^+^ and its metabolites. The molecular mechanisms underlying the Ca^2+^ dependent regulation of O-GlcNAc levels also requires further investigation.

## Data Availability

The raw data supporting the conclusion of this article will be made available by the authors, without undue reservation.
