# Supplementary material for: The Identification of a Novel Calcium-Dependent Link Between NAD+ and Glucose Deprivation-Induced Increases in Protein O-GlcNAcylation and ER Stress
Source: Front Mol Biosci. 2021 Dec 7;8:780865. doi: 10.3389/fmolb.2021.780865 (PMC8691773; doi:10.3389/fmolb.2021.780865)
Supplement: Supplementary file 1 [file DataSheet1.PDF]

Supplementary Figures

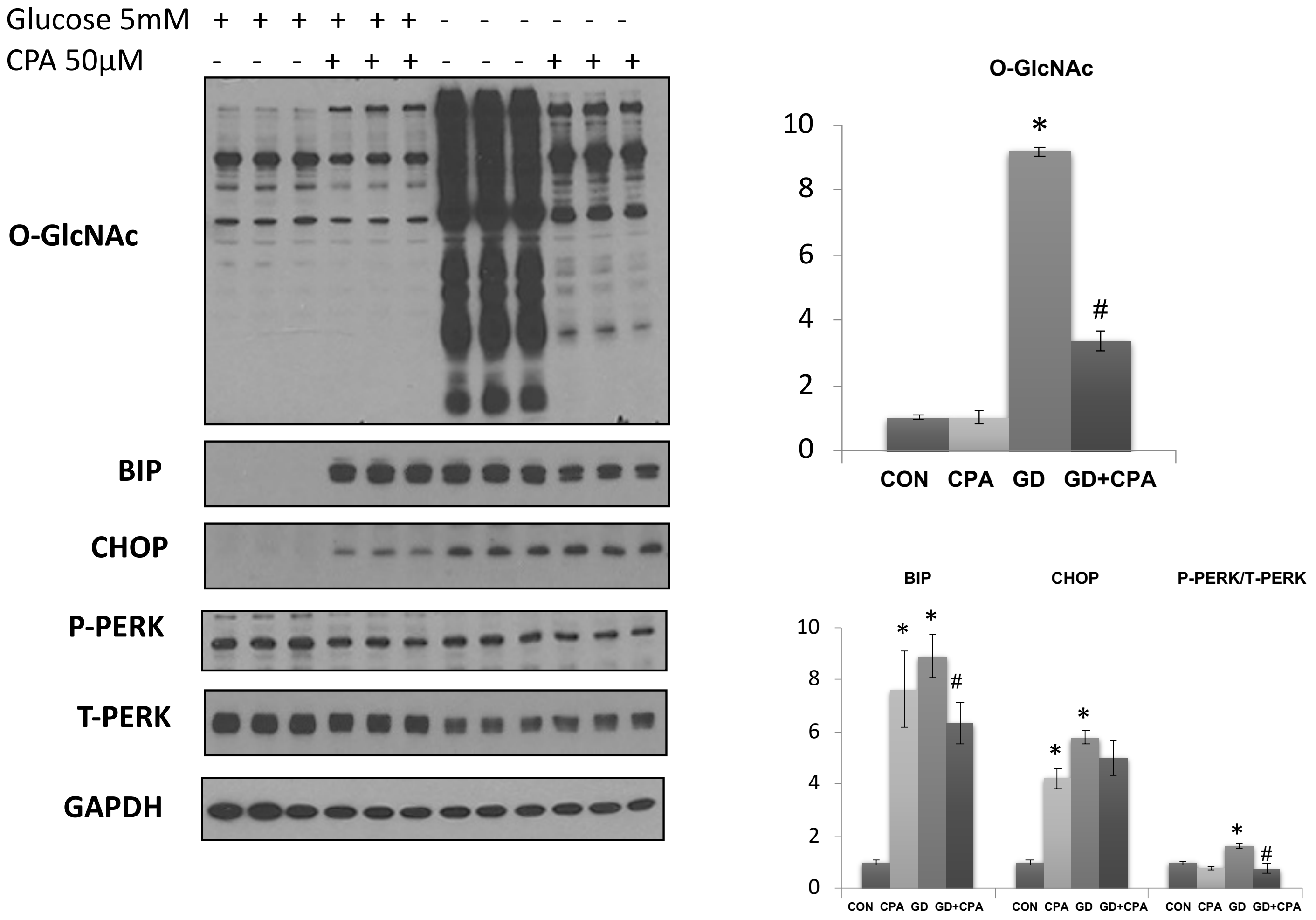

Supplemental Figure 1: **The effects of SERCA inhibitor cyclopiazonic acid (CPA) on the glucose deprivation induced increase in O-GlcNAc levels and ER stress:** Left panel: O-GlcNAc immunoblots with and without glucose in the presence or absence of SERCA inhibitor cyclopiazonic acid CPA (50µM). Right panel Quantification of immunoblots normalized to GAPDH.

Supplement Fig 2

A)

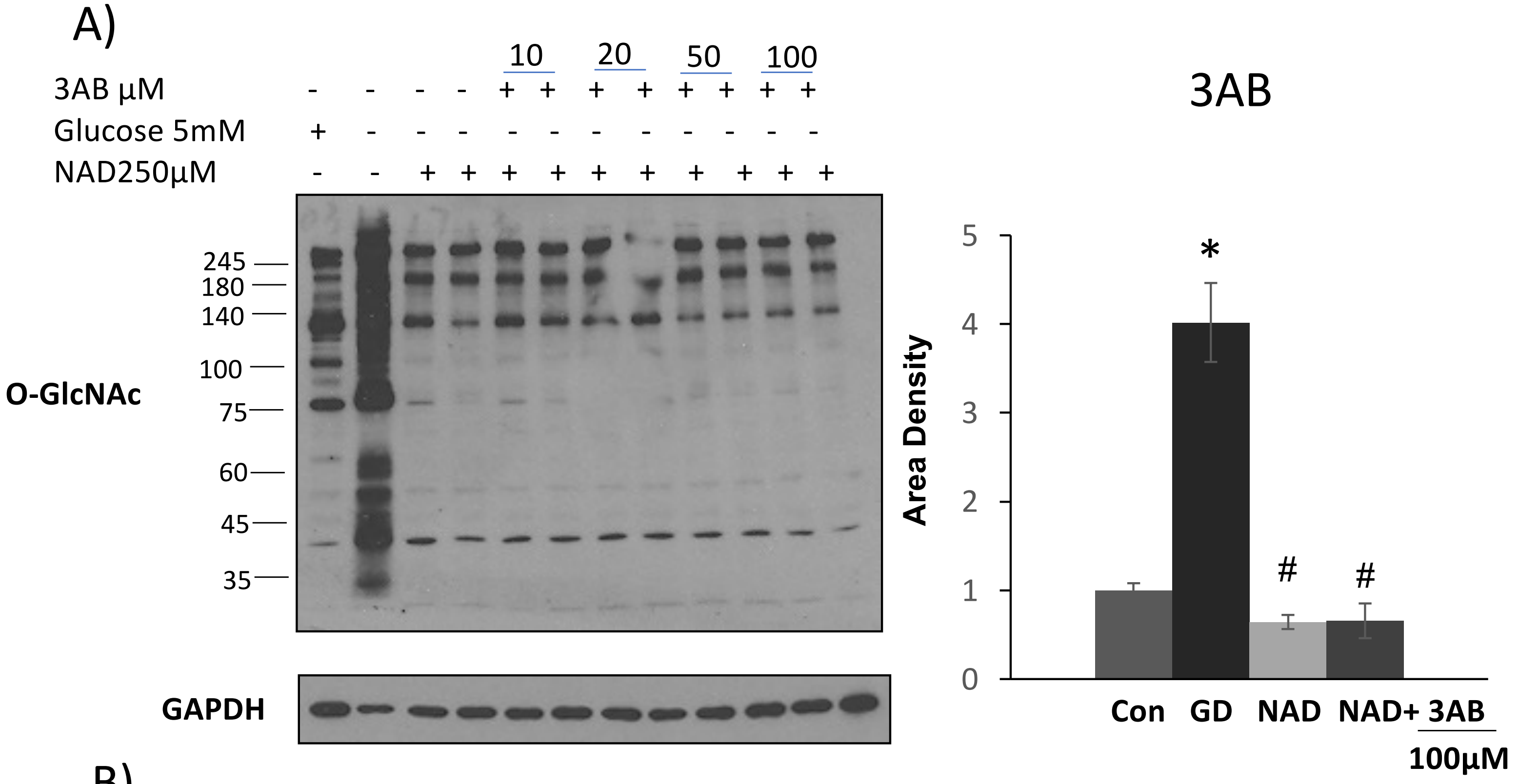

B)

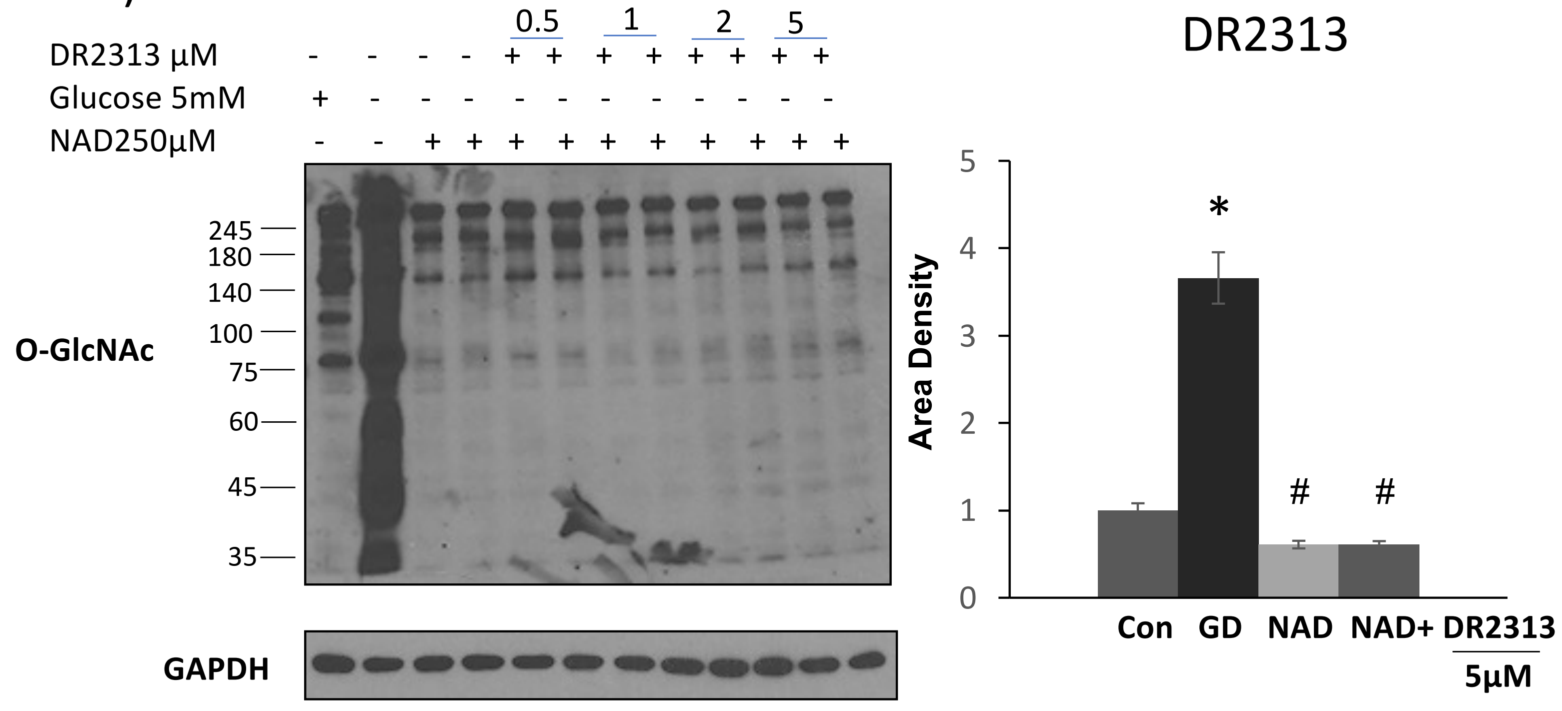

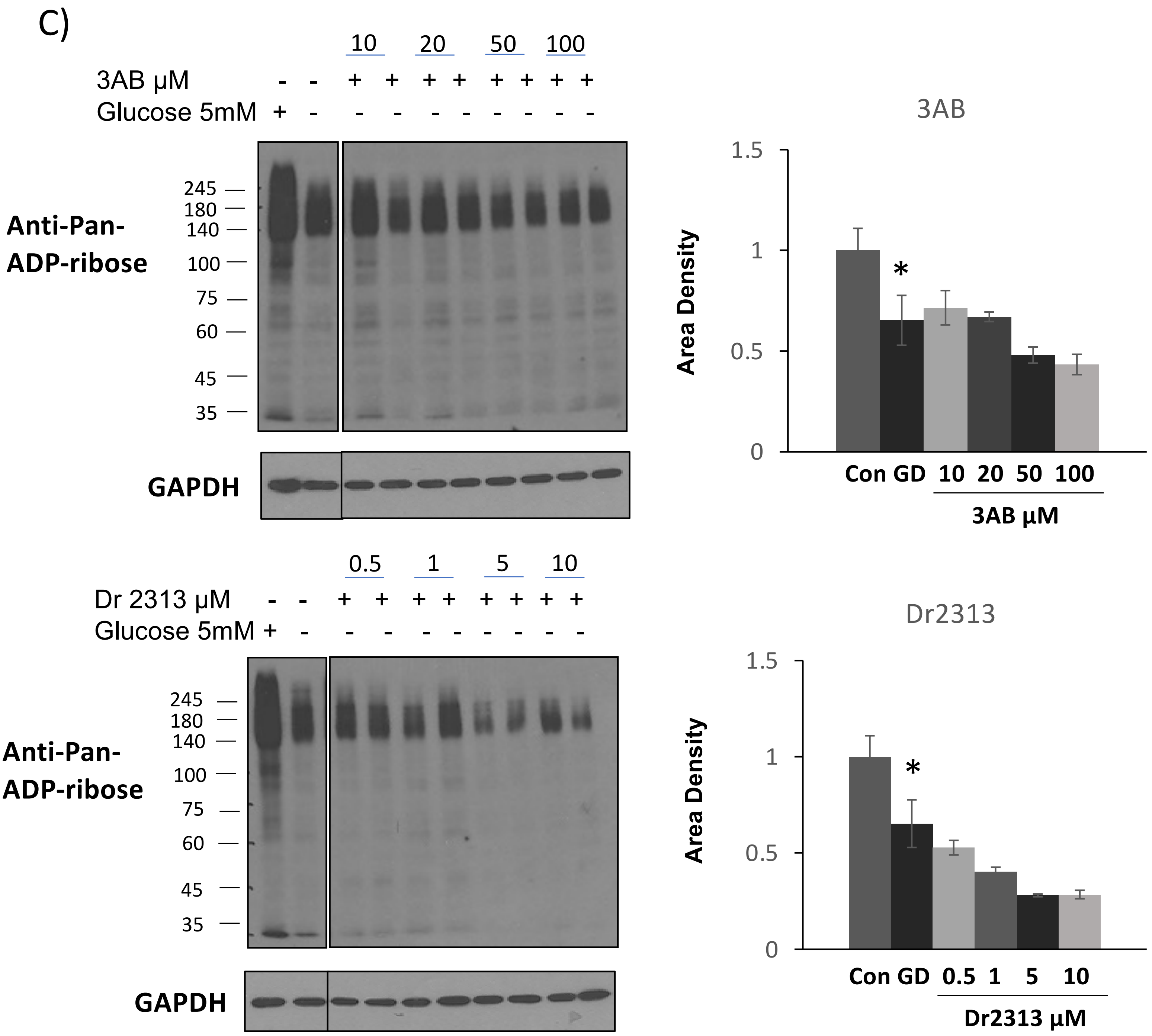

Supplemental Figure 2: **The effects of PARP1 inhibitors 3AB and DR2313 on NAD^+ mediated attenuation of O-GlcNAc in response to glucose deprivation:** A) Left panel: O-GlcNAc immunoblots with and without glucose in the presence or absence of 3AB (10, 20, 50, 100µM) plus NAD^+ (250µM); Right panel Quantification of immunoblots at 100µM 3AB normalized to GAPDH. B) Left panel: O-GlcNAc immunoblots with and without glucose in the presence or absence of DR2313 (0.5, 1, 2, 5µM) plus NAD^+ (250µM); Right panel: Quantification of immunoblots at 5µM DR2313 normalized to GAPDH. C) Left panels: Poly-ADP ribosylation immunoblots in the presence or absence of 3AB (10, 20, 50, 100µM) and DR2313 (0.5, 1, 2, 5µM). Right panels: Quantification of immunoblots normalized to GAPDH. *$p<0.05$ vs Control (Con) group; # $p<0.05$ vs glucose deprivation (GD) group.

.
